# Epilepsy clinic analytics: leveraging EMR reporting tools to characterize center volume and complexity

**DOI:** 10.3389/fneur.2025.1722470

**Published:** 2026-01-12

**Authors:** Vanuli Arya, Arindam Ghosh Mazumder, Muna Nnamani, Mark A. Abboud, Samuel C. Lee, Michael S. Guzman, Vaishnav Krishnan

**Affiliations:** 1 Departments of Neurology, Neuroscience and Psychiatry & Behavioral Sciences, Baylor College of Medicine, Houston, TX, United States; 2 Office of Information Technology, Baylor College of Medicine, Houston, TX, United States

**Keywords:** epilepsy centers, neuromodulation, outpatient clinic, polytherapy, practice management

## Abstract

**Introduction:**

Epilepsy centers connect patients to multidisciplinary provider groups that can coordinate surgical interventions and/or investigational treatments. Automated techniques to cross-sectionally review patient volume and complexity may reveal objective metrics to assign appropriate personnel and resources. Here, we leveraged an electronic medical record (EMR) reporting tool to examine how patient and visit volumes, antiseizure medication (ASM) prescriptions and neuromodulation use evolved over an 11-year epoch at a single level 4 adult epilepsy center in Texas, USA.

**Methods:**

Using Epic Workbench Reporting, we acquired the dates of (i) all clinic visits (office or telemedicine), (ii) CPT codes for neuromodulation interrogation/programming, and (iii) prescriptions for all antiseizure or rescue medications from 14 epilepsy providers between 2013 and 2023. We calculated an annual complexity score (CS) for each patient, incorporating visit frequency and the total number of distinct ASMs prescribed.

**Results:**

Over this 11-year period, the clinic cared for 5,215 unique patients, approximately 20% of whom were only seen once. Our analysis revealed a gradual doubling of total patients served annually, an increase in ASM polytherapy rates and subtle shifts in overall ASM prescribing patterns. High CS were observed in patients treated with neuromodulation and/or administered liquid ASMs. Average annual CS gradually increased over the study period, and many patients displayed wide CS fluctuations over time.

**Discussion:**

Our results validate a simple approach to longitudinally monitor the volume and approximate complexity of patients within an epilepsy center’s ambulatory clinic, providing opportunities to equitably assign personnel and resources, identify candidates for epilepsy surgery and/or remote patient monitoring strategies.

## Highlights

- We illustrate how an electronic medical record (EMR) reporting tool can extract and organize clinical data pertinent to epilepsy care.
- Using such data, we chart the growth and changes in prescribing patterns of a single epilepsy center in Texas USA.
- We devise an annual complexity score to identify patients that may benefit from closer surveillance.

## Introduction

Epilepsy affects approximately 65 million people worldwide (1). Almost 30% of patients with epilepsy (PWE) experience drug-refractory seizures and may benefit from neurosurgical interventions, dietary therapies or access to investigational trials (2). In the United States, expert consideration of these personalized therapeutic strategies typically occurs in epilepsy clinics accredited by the National Association of Epilepsy Centers (NAEC) (3, 4). These include “Level 4” centers, that offer a comprehensive suite of diagnostic and surgical treatment options (5) made possible through multidisciplinary collaborations between epileptologists and neurosurgery (5), neuropsychology, neuroradiology and neuropathology.

For any given epilepsy center, we expect that the volume and complexity of PWE served in that center evolves over time through migration, changes in referral patterns and/or ongoing pediatric to adult transitions of care. Characterizing the clinic population at epilepsy centers can be challenging, on both a center level and at the level of individual patients. Epilepsy disease metrics are often stored in the electronic medical record (EMR) as unstructured data, including key elements such as seizure type, severity, and frequency, brain imaging, electroencephalogram (EEG) changes, and medication response or adverse effects (6). Thus, characterizing patient disease burden and severity can be difficult, and often requires time and labor-intensive manual chart review. There are no rapid or scalable approaches to quickly identifying patients with severe or complex epilepsy from readily available data elements, which can help risk stratify patients who may benefit from additional treatment options, including neuromodulation. Automated techniques to longitudinally monitor clinic size and disease complexity may provide objective metrics to equitably assign personnel and other resources.

Recent studies have explored implementing structured clinical documentation support toolkits in the EMR or utilizing natural language processing using large language models (LLMs) to extract clinical features from unstructured notes (6–8). However, such approaches require significant computational resources and expertise and may not be readily generalizable across institutions (6, 9, 10). With current widespread embrace of EMR systems, several reporting tools have been developed to generate clinical and operational reports with ease. In this study, we utilized one such tool (Workbench Reporting) within the most widely utilized EMR platform (Epic Systems) (11) to visualize trends in overall clinic volume, ASM (antiseizure medication) and neuromodulation use at a single tertiary care epilepsy clinic within a level-IV NAEC Adult Epilepsy Center.

## Methods

### Standard protocol approvals, registrations and patient consents

Study protocols were approved by the Baylor College of Medicine Institutional Review Board (IRB).

### Data extraction

We included patients seen at the adult practice of the Baylor Comprehensive Epilepsy Center between 1/1/2013 and 12/31/2023 by a set of 14 providers (13 epileptologists, 1 epilepsy nurse practitioner). Using EPIC Reporting Workbench tools, we generated: (i) a list of all office or telemedicine visit dates for each patient, (ii) a list of all individual prescriptions for ASMs (including seizure rescue medications) and the dates of those prescriptions, and (iii) dates and entries for CPT codes (current procedural terminology) corresponding to the interrogation/programming of responsive neurostimulation (RNS), deep brain stimulation (DBS) and vagus nerve stimulation (VNS) devices, (encompassing 95,970, 95,974, 95,976, 95,977, 95,975, 95,983, 95,836). Rescue medications were identified as any prescriptions for lorazepam, intranasal midazolam, intranasal diazepam and rectal diazepam. Clonazepam and non-rectal/nasal diazepam were not defined as rescue medications in our analysis as they are frequently prescribed for daily scheduled use, often used as a dual purpose ASM and anti-spasticity agent.

**Figure 1:**
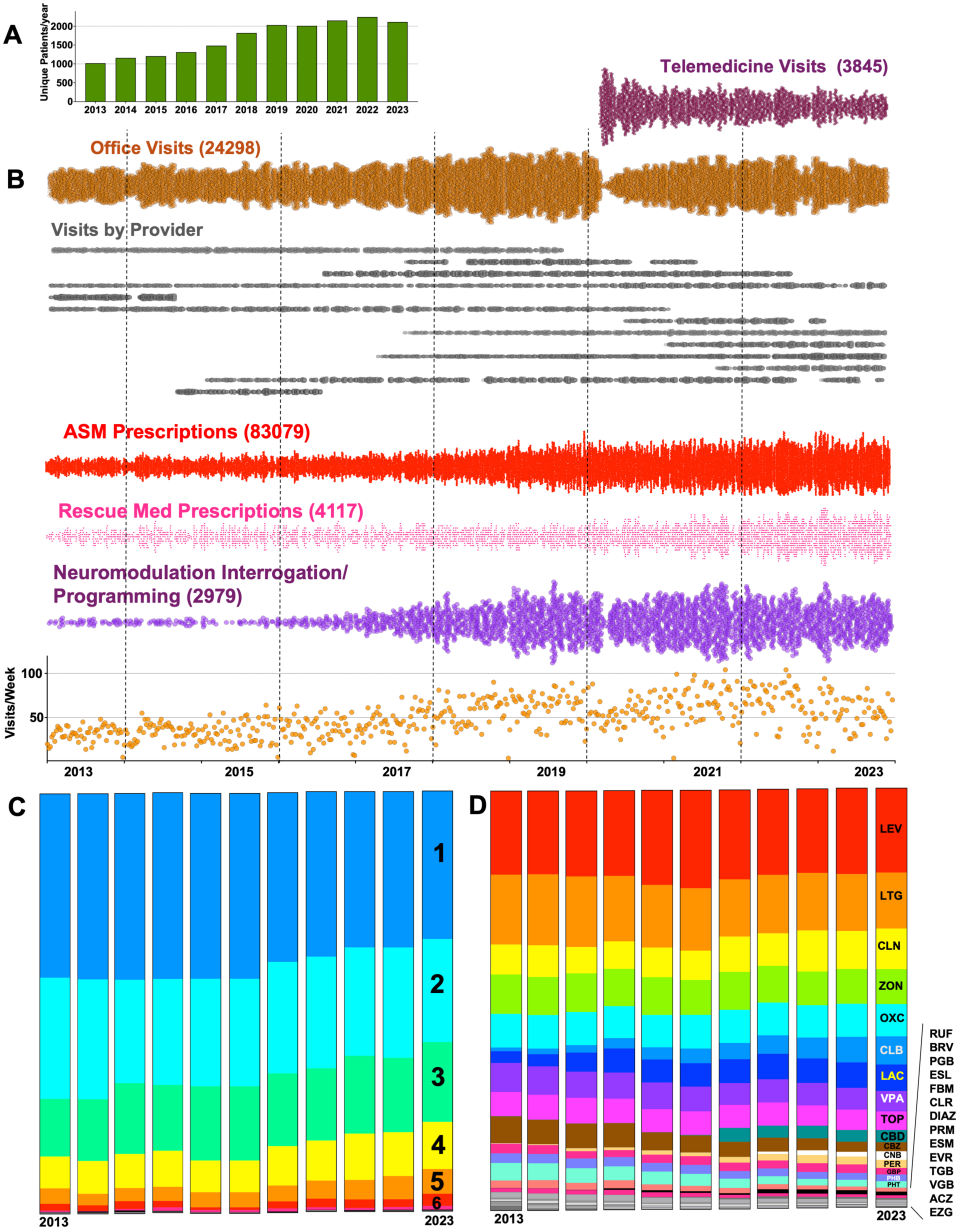
Clinic growth and trends in antiseizure medication (ASM) prescribing captured by EPIC workbench reporting. **(A)** Between 2013 and 2023, the total number of unique patients that were either seen as an office/telemedicine visit *or* received at least one prescription ASM (or rescue medication) approximately doubled from ~1,000 to 2000/year. **(B)** Dot plots capturing the increase in (i) overall office visit frequency, (ii) implementation and embrace of telemedicine visits, (iii) a breakdown of visits by provider, as well as increases in rates of ASM/rescue medications and CPT entries for neuromodulation interrogation/programming (including VNS, DBS and RNS). **(C)** 100% stacked bar graphs showing the proportion of patients (for each calendar year) receiving 1–6 or more distinct prescriptions (including rescue medications). **(D)** 100% stacked bar graph depicting trends in the landscape of ASM prescribing for the same center. LEV, levetiracetam; LTG, lamotrigine; CLN, clonazepam; ZON, zonisamide; OXC, oxcarbazepine; CLB, clobazam; LAC, lacosamide; VPA, valproate; TOP, topiramate; CBD, cannabidiol; CBZ, carbamazepine; CNB, cenobamate; PER, perampanel; GBP, gabapentin; PHB, phenobarbital; PHT, phenytoin; RUF, rufinamide; BRV, brivaracetam; PGB, pregabalin; ESL, eslicarbazepine; FBM, felbamate; CLD, clorazepate; DIAZ, diazepam; PRM, primidone; ESM, ethosuximide; EVR, everolimus; TGB, tiagabine; VGB, vigabatrin; ACZ, acetazolamide; EZG, ezogabine.

For each patient that received at least one prescription ASM or rescue medication for any given calendar year, we tallied and calculated the total number of *distinct* ASMs or rescue medications prescribed for that same year (*Rx*^year^). Immediate release and delayed release tablet formulations of the same ASM were not considered distinct prescriptions. In contrast, tablet and liquid formulations of the same ASM *were* considered distinct, so as to capture the occurrence of a change from tablet/capsule to oral solution formulations. For example, a patient receiving prescriptions for zonisamide and pregabalin in May 2018, who then receives a refill for pregabalin and a switch to liquid zonisamide in December 2018, has an *Rx*^2018^ = 3 (PGB, ZON, ZON-L). To visualize trends in polytherapy, we graphed the proportion of patients by *Rx* for each calendar year as a 100% stacked bar graph (Figure 1C). To similarly visualize interannual shifts in ASM prescription patterns (Figure 1D), we stacked the overall proportions of prescriptions for each ASM (regardless of formulation). Thus, in 2023, prescriptions for levetiracetam and lamotrigine (of any formulation) amounted ~20.2% and ~13.3% of all ASM prescriptions, respectively.

Next, using the same automatically generated visit and prescription data, we sought to devise a metric that would approximate the complexity of a patient’s epilepsy, as reflected by patterns of clinic utilization and/or requirements for polytherapy or changes in ASM therapy. For every patient served between 2013 and 2023, we tallied the total number of visits per year (*V*^year^, office or telemedicine visits) in addition to *Rx*^year^. A single annual complexity score (CS) for every patient was calculated as follows:


$${\text{Complexity score}}^{\text{year}}=[(V^{\text{year}}+1)\;x\;(Rx^{\text{year}}+1)–1]$$

**Figure 2:**
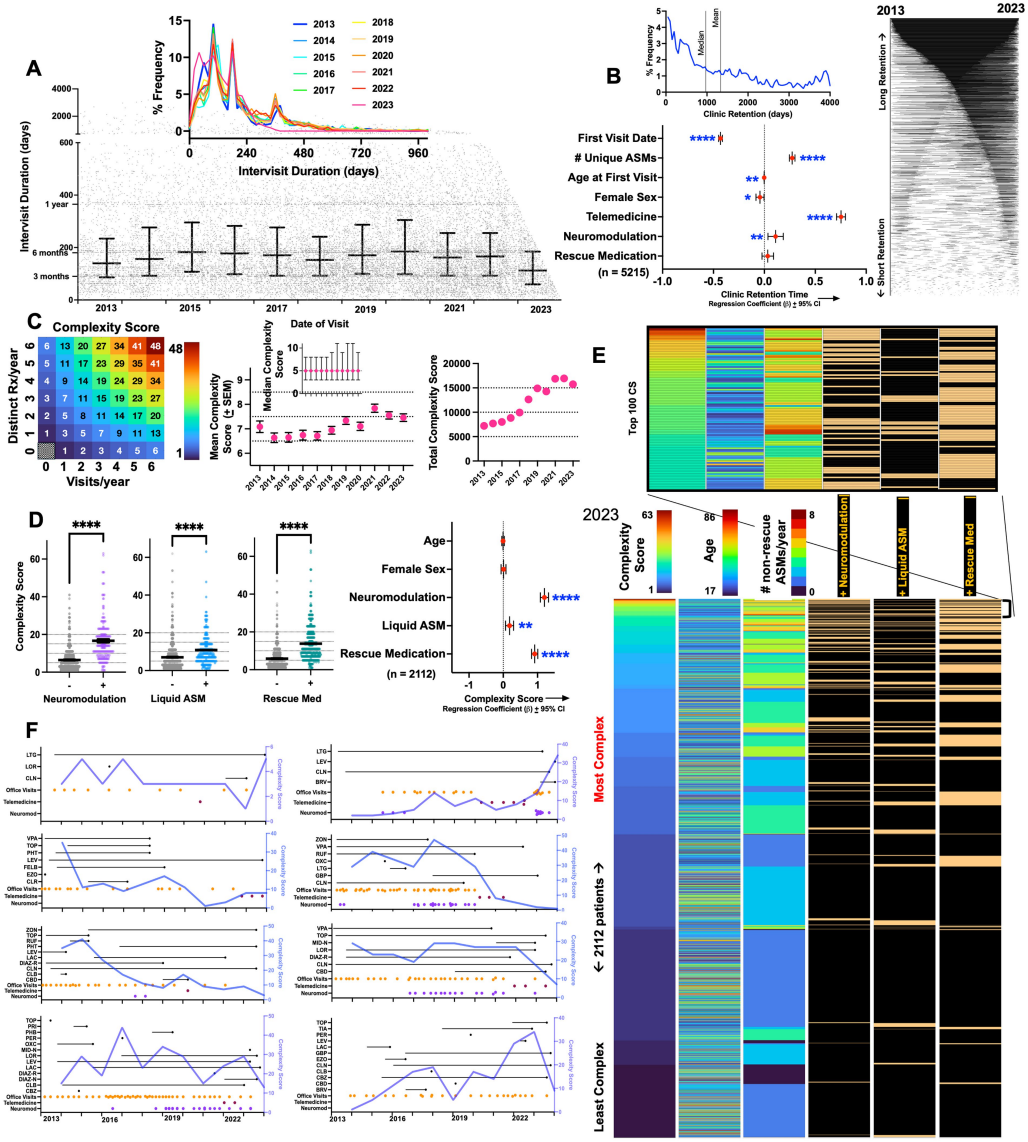
Epilepsy complexity scores (CS). **(A)** Scatter plot depicting appointment date (x-axis) and the # of days until the next appointment (*y*-axis). Error bars depict median ± interquartile range. Inset: annualized frequency distribution histograms of intervisit durations, capturing peaks of follow up durations corresponding to ~90, 180, and 360 days in duration (*x*-axis is capped at 1000). **(B)** Frequency distribution histogram of all non-zero clinic retention durations (defined as the duration between the first and last visit date). Right: retention durations for 4,067 patients depicted as lines connecting first and last visit dates. Beta coefficients and 95% confidence intervals for a multiple linear regression model examining predictors of long-term clinic retention. **(C)** Illustration to visualize how CS varies by changes in visit frequency and ASM prescriptions. Mean CS increased from ~6.6–7 (in 2013 and 2014) to ~7.5 (in 2022 and 2023). Error bars depict standard errors of the mean (SEM). Inset shows median CS per year ± interquartile range. **(D)** In 2023, CS were significantly higher in patients receiving neuromodulation, liquid ASMs, or prescriptions for rescue medications (Welch’s *T* tests). Beta coefficients ± 95% confidence intervals shown for a multiple linear regression model examining predictors of high complexity scores. **(E)** Dashboard of a total of 2,112 patients that were either seen as a visit or received an ASM prescription in 2023, ranked by descending CS. Data is shown as a heatmap where every patient is depicted on a separate row. Inset captures patients with the top hundred CS. **(F)** Changes in complexity scores visualized over time in eight representative patients, annotated with visit dates as well as start and end dates for ASM/rescue medications. *, **, ***, and **** depict *p* < 0.001 and 0.0001, respectively. LEV, levetiracetam; LTG, lamotrigine; LOR, lorazepam; CLN, clonazepam; ZON, zonisamide; OXC, oxcarbazepine; CLB, clobazam; LAC, lacosamide; VPA, valproate; TOP, topiramate; CBD, cannabidiol; CBZ, carbamazepine; CNB, cenobamate; PER, perampanel; GBP, gabapentin; PHB, phenobarbital; PHT, phenytoin, RUF, rufinamide; BRV, brivaracertam; PGB, pregabalin; ESL, eslicarbazepine; FBM, felbamate; CLD, clorazepate; DIAZ, diazepam; PRM, primidone; ESM, ethosuximide; EVR, everolimus; TGB, tiagabine; VGB, vigabatrin; ACZ, acetazolamide; EZG, ezogabine; MID-N, intranasal midazolam; DIAZ-R, rectal diazepam; DIAZ-N, intranasal diazepam.

A constant offset of +1 was added to both *V*^year^ and *Rx*^year^ to ensure that all values were strictly positive, and a final offset of −1 assigned a score of zero to patients who were neither seen nor prescribed any ASMs in a given year. Zero values were excluded from the calculation of mean annual complexity scores (Figure 2C).

Thus, a patient that receives a prescription for carbamazepine and perampanel in March of 2017, and then is prescribed liquid carbamazepine, perampanel, levetiracetam and intranasal midazolam at a second visit in November 2017, is assigned a CS of (2 + 1)^visits^ * (4 + 1)^ASMs^ – 1 = 14 (see Figure 2C).

Statistical Analysis: Microsoft Excel and MATLAB functions were generated to parse data and calculate ASM start and end-dates. Prism Graphpad 10.4 was utilized to create graphs and perform multiple linear regression (Figure 2B) and Welch’s *T* tests (Figure 2C). Variance inflation factors pertaining to the covariates included in regression models were always ≤ 4.

## Results

Between 2013 and 2023, the clinic cared for 5,215 unique patients. 1,148 patients were seen only once (either as an office or telemedicine visit) and 896 patients were seen without ever receiving a prescription or neuromodulation interrogation/programming (689 patients overlapped between these two subgroups). As shown in Figure 1A, the total number of unique patients cared for annually approximately doubled during the observation period, from 1,019 to 2,112 patients. This growth was accompanied by an increase in total visits, ASM (and rescue) prescriptions and CPT entries for neuromodulation (Figure 1B). As the COVID-19 pandemic necessitated remote clinical care, telemedicine visits were instituted in ~April 2020. A transient pause in neuromodulation interrogations/programming was observed, but prescriptions for ASMs and rescue medications robustly endured without interruptions.

The percentage of patients receiving only a single *distinct* (see Methods) prescription per year (ASM or rescue) reduced from ~43% (2013) to 35% (2023, Figure 1C), signifying a trend towards caring for a patient population that is increasingly dependent on polytherapy. Among non-rescue ASMs, there were subtle shifts in prescribing patterns. This included a gradual surge in shares of prescriptions for clobazam, lacosamide and perampanel, with concomitant reductions in the use of carbamazepine and phenytoin. An abrupt increase in cannabidiol prescriptions was evident in 2019, with a more gradual increase in prescriptions for cenobamate seen following 2020. Across all years, levetiracetam and lamotrigine together constituted 35–40% of all ASMs prescribed.

We next explored visit frequencies and patterns of patient retention within the clinic. As shown in Figure 2A, we observed a spectrum of intervisit durations [days between two consecutive appointments (virtual or office)], with peaks observed at ~3, 6 and 12 months (median of ~140–160 days). This distribution remained largely unchanged between 2013 and 2023. Among patients that were seen at least twice, we defined clinic retention as the duration between the very first and last visit (occurring between 2013 and 2023). Median retention times were ~970 days (mean: 1335 days, Figure 2B). As expected, multivariate analysis showed that retention times were greater in patients that had an earlier first visit date. Retention was also positively associated with the total number of unique ASMs prescribed, telemedicine engagement and the use of neuromodulation. Female sex and a younger age first visit also negatively correlated with retention, albeit with more miniscule effect sizes.

Using prescription data and visit dates, we calculated annual complexity scores (CS, see Methods) for each patient seen every year. Mean CS within the center grew from ~6.5 (in 2013) to ~7.5 in 2023 (Figure 2C). A CS of 7 is equivalent to approximately three visits and one ASM/year (or one visit and three unique ASMs/year). The center’s median CS remained stable at 5 throughout, while the center’s total annual CS tracked annual patient volumes (Figure 1A). Among patients seen in 2023, we examined how CS correlated with other surrogate metrics of epilepsy complexity (Figure 2D). CS were significantly higher in patients receiving neuromodulation (who are currently or have in the past experienced medically intractable seizures), in patients prescribed rescue medications (often reserved for those with drug-refractory seizures that can be either prolonged or cluster (12)), and marginally higher in those receiving liquid ASMs [a surrogate of dysphagia or gastrostomy dependence (13, 14), observed in intellectually disabled patients whose epilepsy tends to be more severe (15, 16)]. This was also confirmed in multivariate analysis (Figure 2D), where the use of neuromodulation, liquid ASMs and rescue medication use were independently associated with a high complexity score, while age and sex were not significant predictors.

We next leveraged complexity scores to survey the center’s 2023 patient population by creating a dashboard of all 2,112 patients seen in that year, ranked by descending CS (Figure 2E). As expected, patients at the top of this list were frequently recipients of ASM polytherapy, neuromodulation and rescue medications. While mean complexity scores across the practice stayed constant, complexity scores within subjects often varied widely. In Figure 2F, we visualize how complexity scores varied over time in eight patients in relation to ASM prescriptions and visit dates, capturing the dynamic range and within-subject fluctuations of this metric.

## Discussion

In this study, we illustrate how a clinical reporting tool implemented within a widely utilized EMR platform can provide a swift and straightforward assessment of the growth and clinical complexity of patients cared for by a representative Level IV NAEC epilepsy center. Importantly, our approach did not require manually ‘opening’ or entering a single chart, leveraging only readily available data elements. To putatively rank patients by complexity, we designed a complexity score (calculated annually) that incorporated measures of ASM polytherapy and visit frequency (including both office and telemedicine visits). ASM polytherapy in epilepsy has been linked with poorer quality of life (QOL) and an increased risk of adverse events (17). On average, patients receiving a rescue ASM, liquid ASMs, or neuromodulation therapy had significantly higher complexity scores. While this approach does not directly measure seizure frequency or definitively identify patients with drug-refractory seizures, it provides an initial scaffold for identifying high risk cohorts of patients that may be triaged to earlier follow up visits, and who may benefit from targeted interventions to improve QOL, including closer follow up visits, proactive care planning, remote patient monitoring strategies and/or neurosurgical interventions. Similarly, improvements in complexity scores over time may support transitions to lower-acuity care settings, helping to optimize resource allocation at epilepsy centers. Our strategy can be similarly implemented and compared across Level IV adult epilepsy centers, providing opportunities for improvements in overall care quality, resource allocation and operational efficiency.

The longitudinal data we present reveal a set of interesting trends. First, we found that clinic providers and patients were able to rapidly embrace telemedicine visits at the onset of the COVID-19 pandemic. By 2023, telemedicine encounters constituted ~25% of all visits in the epilepsy clinic. Second, between 2019 and 2023, we observed a gradual surge in the proportion of patients that received two or more ASMs (including rescue medications), increasing from ~60 to 65%, aligned with the gradual increase in mean complexity scores seen during this period. The most frequently prescribed ASMs remained largely constant, and included levetiracetam, lamotrigine, zonisamide and oxcarbazepine. However, there were changes in the utilization of other ASMs, including increases in clobazam, lacosamide, cannabidiol, perampanel and cenobamate, with concomitant reductions in the use of carbamazepine and phenytoin. These data speak directly to the malleability of center providers to accept newer ASMs, especially those with novel mechanisms of action. Third, we showed that despite increases in clinic volume, intervisit durations remained largely stable, made possible in part by increasing the number of providers (4–5 in 2013–2015, to 7–10 in 2021–2023). Fourth, at least one in five patients were only ever seen once: these may represent patients with new onset seizures and/or those seeking a second opinion. On average, patients remained within the clinic for approximately 1,335 days (~3.6 years). After correcting for earliest visit date (follow up opportunity bias), longer retention times were observed in patients that utilized telemedicine visits and who were prescribed a higher number of ASMs. Telemedicine availability often facilitates follow-up for patients who reside at greater distances or face logistical barriers to in-person care, which may partially explain the strong association between telemedicine use and longer retention.

Xie et al. (8) recently published an alternative strategy to longitudinally survey an epilepsy center’s patient population. Here, they used natural language processing (NLP) algorithms to parse through 55,000 unstructured clinic notes from ~9,000 patients over a 13-year period, to extract information about seizure freedom, seizure frequency and the date of the most recent seizure. Data pertaining to ASM use and/or neuromodulation was not incorporated into their algorithms. Using this technique, they revealed that most patients at their center had a mixture of seizure-free and recent-seizure visits, with highly variable seizure free-intervals. Our analysis found similar fluctuations in complexity scores over time, reflecting the waxing and waning nature of disability and/or healthcare utilization in persons with epilepsy.

We acknowledge several limitations. First, complexity scores measured in our study should be interpreted cautiously as an operational metric that only reflects (i) a need for ASM polytherapy (or frequent changes in ASM therapy) and/or (ii) visit frequency/clinic utilization. We did not incorporate other surrogate measures of epilepsy complexity or severity, such as seizure frequency/refractoriness, emergency room visits, epilepsy monitoring unit admissions, EEG/MRI findings or comorbid conditions. Increases in CS driven solely by increases in visit frequency may reflect true increases in epilepsy severity (such as worsening seizure burden or ASM side effects), but may be artifactually driven by other explanations, such as patient insistence or patient-specific improvements in clinic compliance without changes in seizure burden. The initiation of neuromodulation therapies also typically requires several short-interval visits for device programming/titration in the early post-implant period. These visits would be expected to increase CS, without necessarily reflecting a true worsening of epilepsy severity. Second, our approach also assumes that prescriptions were in fact filled in a timely fashion and does not take insurance delays or denials into account. Third, we do not resolve the indication for a prescription (e.g., topiramate for migraines, epilepsy or both). Fourth, our data does not include persons with epilepsy who were cared for by a distinct group of general neurologists within the department. Future iterations of complexity scores may benefit from integrating NLP approaches with integrated data from electronic health records outside of the center’s main clinic EMR using health information exchange features (e.g., Care Everywhere).

In conclusion, we present a simple and efficient tool to longitudinally monitor the demography of an epilepsy center’s patient population (and compare these metrics across centers). We provide one strategy (complexity scores) to cross-sectionally rank patients by the complexity of their clinic utilization and polytherapy burden, identifying candidates who may benefit from remote surveillance programs and/or adjunct surgical therapies.

## Data Availability

The data analyzed in this study is contained within our EMR. Requests to access these datasets should be directed to VK, vkrish@Bcm.edu.
